# Dietary Intake Nutritional Status and Lifestyle of Adolescent Vegetarian and Nonvegetarian Girls in New Zealand (The SuNDiAL Project): Protocol for a Clustered, Cross-Sectional Survey

**DOI:** 10.2196/17310

**Published:** 2020-05-27

**Authors:** Meredith Peddie, Chaya Ranasinghe, Tessa Scott, Anne-Louise Heath, Caroline Horwath, Rosalind Gibson, Rachel Brown, Lisa Houghton, Jillian Haszard

**Affiliations:** 1 Department of Human Nutrition University of Otago Dunedin New Zealand

**Keywords:** vegetarianism, teenagers, women, iron, zinc, calcium, B12, physical activity, attitudes motivations, beliefs

## Abstract

**Background:**

Anecdotally, vegetarian eating patterns seem to be increasing in parallel with growing concerns about environmental sustainability. While this pattern of eating is widely believed to be associated with benefits for the planet and individual health, it may increase the risk of inadequate intakes and nutrient deficiency if not planned carefully. Adolescent girls may be particularly at risk, as they have increased requirements for nutrients such as iron, zinc, calcium, and vitamin B12 during growth and development.

**Objective:**

The objective of the SuNDiAL Project (Survey of Nutrition, Dietary Assessment, and Lifestyles) is to compare the dietary intakes and habits, nutrition status, motivations, attitudes, and physical activity of a sample of vegetarian and nonvegetarian adolescent girls in New Zealand.

**Methods:**

A clustered, cross-sectional, nationwide study of adolescents aged 15-18 years was conducted. Secondary schools were recruited throughout New Zealand, and pupils (n=290) were invited to participate in data collection in either the first (February to April) or third (August to October) school term of 2019 (New Zealand schools operate on a 4-term year). Sociodemographic and health information; vegetarian status; dietary habits; and attitudes, motivations, and beliefs regarding food choices were assessed via an online self-administered questionnaire. Dietary intakes were collected via two 24-hour diet recalls on nonconsecutive days and will be adjusted for within-person variation using the Multiple Source Method, to represent usual intakes. Nutrient adequacy will be assessed by the estimated average requirement cut-point method or probability approach as appropriate. Height and weight were measured, and blood and urine samples collected for micronutrient status assessment. Participants wore an accelerometer for 7 days to assess 24-hour activity patterns (time spent asleep, sedentary, or engagement in light-intensity or moderate-to-vigorous intensity physical activity).

**Results:**

Recruitment and data collection were conducted in 2019. Data are currently being cleaned and analyzed, with publication of the main results anticipated at the end of 2020.

**Conclusions:**

The SuNDiAL Project will provide a meaningful and timely description of diet, nutrition status, and motivational factors associated with vegetarianism and identify any risks this pattern of eating may pose for female adolescents. The results of this study will support the development of targeted recommendations and interventions aimed at enhancing the health, growth, and development of adolescent girls.

**Trial Registration:**

Australian New Zealand Clinical Trials Registry ACTRN12619000290190; https://tinyurl.com/yaumh278

**International Registered Report Identifier (IRRID):**

DERR1-10.2196/17310

## Introduction

### Background

Few, if any, robust estimates of the prevalence of vegetarianism in populations exist, although plant-based and vegetarian diets (defined as not consuming any red meat, poultry, or seafood for the purposes of this article) appear to be growing in popularity. Increasing concern regarding the importance of environmental sustainability may explain this apparent rise; however, health is reported as a significant motivator for many vegetarians [1]. Indeed, vegetarians tend to have a body mass index that is 1-2 kg/m^2^ lower than their otherwise comparable nonvegetarian peers and exhibit less weight gain during adulthood [2]. Vegetarians also have a slightly lower risk of some cancers [2] and as much as a 24% lower risk of ischemic heart disease [2], presumably because they tend to have lower total and low-density lipoprotein cholesterol concentrations [2]. However, much of the data that underpin our understanding of how vegetarianism may affect disease incidence was collected from adult populations prior to the 1990s [3,4] or in the early 2000s [5]. Much less is understood about the foods and nutrient intake of vegetarian adolescents. Recent advances in food technology, food fortification, and the widespread availability of products designed to be plant-based substitutes for meat and milk imply that vegetarians can now choose from many commercially produced food products [6]. However, consistent with older research, more recent studies indicate that vegetarian or vegan eating patterns score higher on the healthy eating index due to a lower sodium and saturated fat intake and higher intakes of fruits and vegetables [7].

A well-planned vegetarian diet containing vegetables, fruits, whole grains, legumes, nuts, and seeds can provide adequate nutrition for most members of the population [8]. In general, vegetarian diets provide large amounts of phytate, dietary fiber, folate, vitamins C and E, and magnesium, but without planning, they may have low protein, vitamins D and B12, iron, zinc, and calcium (particularly among vegans who do not consume animal products of any kind) [8,9]. Additionally, while the iron and zinc content of a vegetarian diet may be similar to that of a nonvegetarian diet, the high phytate content, absence of heme iron from cellular animal sources, and lower animal protein intake reduces the bioavailability of iron and zinc, significantly increasing the risk of deficiency [10]. The risk of vitamin B12, calcium, iron, and zinc deficiency may be greater in certain sex and life-stage groups such as young female adults [10-12]. The pubertal growth spurt, sexual maturation, and the onset of menarche increase requirements for vitamin B12, calcium, iron, and zinc in adolescent girls [12]. Increased autonomy over food intake and reductions in energy intake due to a desire to lose weight or achieve a certain body type may further contribute to the risk of nutrient deficiencies in this age group that could be exacerbated in vegetarians without careful food choice. Indeed, the latest representative data collected in New Zealand over a decade ago indicates that 88% of female adolescents have inadequate intakes of calcium, and 34% have inadequate intakes of iron, with 11% being identified as having iron deficiency and a further 5% as having anemia [13], despite the estimation that less than 9% of this age group was likely to be vegetarian at the time of data collection [14].

Reasons for following a vegetarian diet include health [1], ethical and environmental concerns [1,15,16], animal welfare [1,15,16], and religious beliefs [16]. However, adolescent girls in New Zealand, who are already at risk of low calcium intakes and iron status, may be further increasing that risk if they do not follow a carefully planned vegetarian diet. It is crucially important to develop guidelines that mitigate the risk and maximize potential benefits, such as reducing saturated fat and increasing fiber intakes. Understanding the motivations, attitudes, and beliefs that underpin food choices is important to inform the development of appropriate and effective guidelines, in particular, to understand why some people choose to be vegetarian and others do not. Other lifestyle behaviors, such as physical activity, that may go hand-in-hand with food choices, should also be examined in relation to health risks and benefits of a vegetarian diet. This knowledge can then be used to appropriately and effectively communicate lifestyle recommendations for those following a vegetarian diet.

### Objectives

Anecdotally, vegetarianism appears to be increasing in popularity. While this increase in popularity may confer some health benefits to the population, adolescent girls in New Zealand are already at an increased risk of inadequate intakes of iron and calcium, which would clearly be exaggerated if animal products were avoided. A poorly planned vegetarian diet can increase the risk of some nutrient deficiencies that may be exacerbated in female adolescents. Therefore, it is critical that the nutrient intake and status of vegetarian adolescent girls are assessed. Furthermore, assessing motivations, attitudes, and beliefs will further our understanding of dietary choices and inform the development of health promotion materials and programs targeted to this age group.

The aim of the SuNDiAL Project (Survey of Nutrition, Dietary Assessment and Lifestyles) is to compare the dietary intakes and habits, nutrition status, motivations, attitudes, and physical activity of a sample of vegetarian and nonvegetarian adolescent girls in New Zealand. The objectives of this study are to describe and compare the following between vegetarians and nonvegetarians:

- Dietary intakes of macronutrients, free and added sugars, phytate, fiber, and key micronutrients (iron, zinc, vitamin B12, folate, iodine, and calcium)
- Biochemical status of key micronutrients (iron, zinc, vitamin B12, and folate)
- Attitudes toward and motivations for food choice (eg, the environment, animal welfare, health)
- Twenty-four–hour activity patterns (sleep, sedentary behavior, and physical activity)
- Dietary habits
- Weight loss intentions

## Methods

### Study Design

The SuNDiAL project is a nationwide cross-sectional survey of female adolescents aged 15-18 years. Nationwide data collection was achieved by utilization of a cohort of postgraduate research students. At the University of Otago, second-year Master of Dietetics students are required to undertake a 6-month research project in addition to 6 months of clinical placement. These student researchers are trained in dietary assessment and clinical skills, making them ideal data collectors for this study. In groups of 2-4 students, they collected data in locations convenient to their clinical placement or home city in New Zealand. In total, data were collected in 8 locations throughout New Zealand. The goal was to recruit at least one secondary school in each of Dunedin, Christchurch, Wanaka, Nelson, Wellington, Tauranga, Whangarei, and New Plymouth. These locations cover a range of cities from small (Wanaka) to large (Christchurch), from the south (Dunedin) to the north (Whangarei) of New Zealand. Data were collected in the first (February to April) or third (August to October) term of school in 2019 (New Zealand secondary schools operate on a 4-term year). The underlying ethnic makeup of the female population aged 15-18 years living in these areas is 70% New Zealand European, 17% Māori, 8% Pacific, and 15% Asian. Socioeconomic status information for this age group is not readily available in New Zealand. However, because we used a convenience sample, the final study population may differ from the overall population. This study has been approved by the University of Otago Human Ethics Committee (Health) (H19/004) and is registered with the Australian New Zealand Clinical Trials Registry (registration number: ACTRN12619000290190). Informed consent was obtained electronically from all participants via an online questionnaire [Research Electronic Data Capture (REDCap), production server version 9.3.3]. In addition, parental consent was obtained via email for participants who were under 16 years of age.

### Selection of Schools

**Figure 1:**
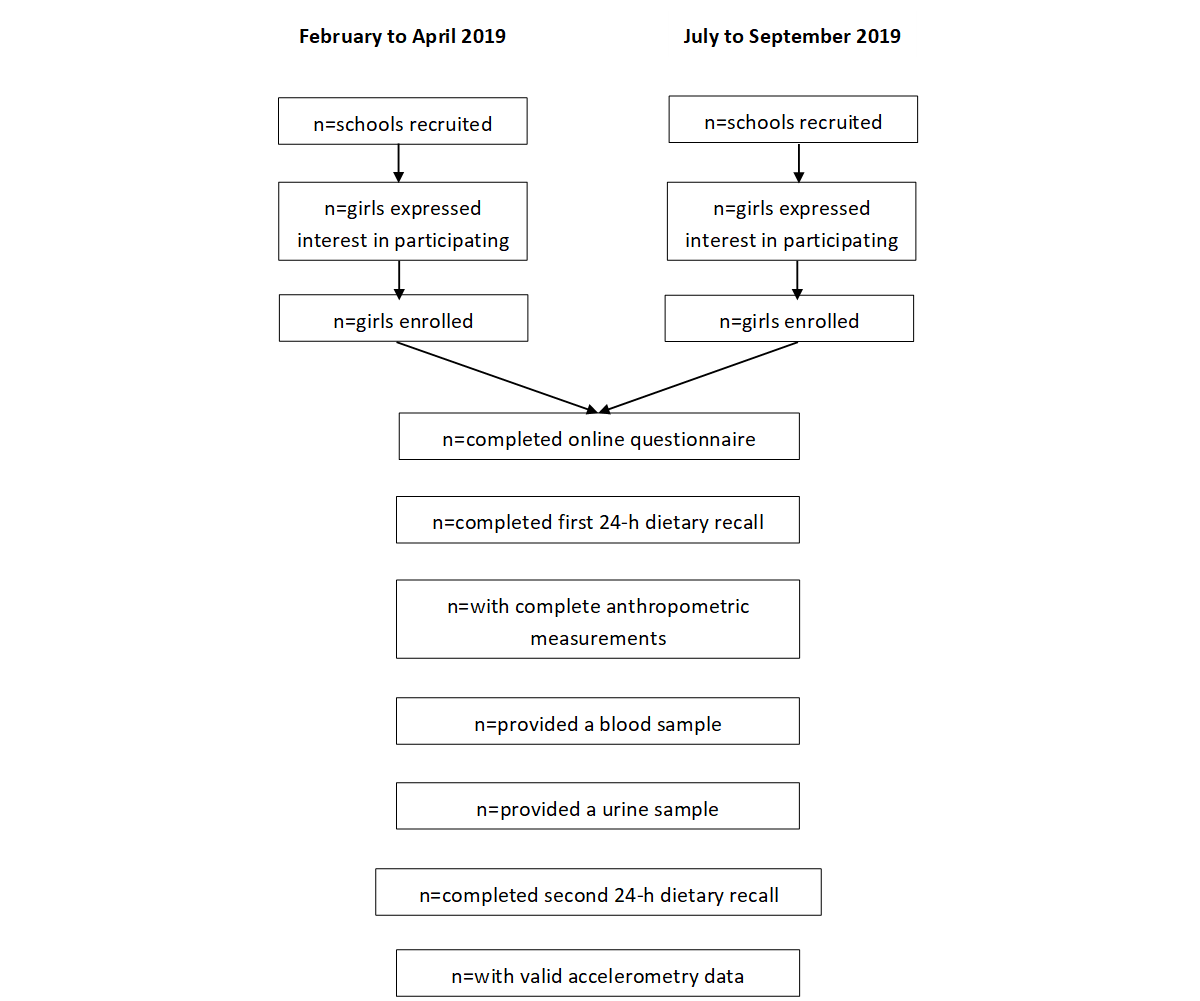
Study Design.

Initially, secondary schools in the predetermined locations were selected to be invited to participate. Initial selection was made by selecting 2-5 schools per location, with a female roll number of at least 200. Lower decile schools (a measure of the socioeconomic status of the school) were preferentially selected for this round of invitation to ensure representation (Figure 1). The selected schools received emails and follow-up phone calls inviting them to participate. If the required number of schools was not reached through this method, other schools in the area were contacted and invited to participate. Schools that were interested provided written consent to participate (signed by an appropriate representative from the school).

### Recruitment of Participants

A brief information session (10-15 min) was delivered by the Master of Dietetics students to eligible pupils at each consenting school. At the session, pupils were given detailed information about the study and the required commitment. An expression of interest form was distributed for individuals to indicate their interest in participating by providing their email address. Individuals were also able to indicate interest on the study website [17]. Electronic and print information about the study was distributed at the school for anyone who required further information about the study. Individuals who provided their email address were sent a link to an online questionnaire (hosted on REDCap), on which they completed consent forms and answered a series of sociodemographic and general health questions. Participants who were under 16 years of age were asked to provide a parent’s email address and did not receive the link to the online questionnaire until a parent or guardian had consented to their participation.

### Inclusion Criteria

Adolescents who identify as female, are aged between 15 and 18 years, are enrolled in one of the selected secondary schools, can speak and understand English, and are not pregnant were eligible to participate.

### Sample Size

A sample size of 300 adolescent girls from 13 secondary schools will have 80% power to the *P*=.05 level to detect a 0.5 standard deviation difference (a “moderate” difference) in continuous outcome variables between vegetarians and nonvegetarians, assuming a prevalence of vegetarianism of 20% and a design effect (for school clusters) of 1.5. If the prevalence of vegetarianism is much less than 20%, then purposeful sampling of vegetarians was planned in the second half of the recruitment year.

### Outcome Measures

#### Online Questionnaire

Once participants completed enrollment, they were asked to complete an online questionnaire that follows from the enrolment questions on REDCap. This questionnaire is divided into three sections. The health and demographics section consists of 29 questions about sociodemographic characteristics and health status including current menstrual status, and food allergies or intolerances. It also asks participants if they identify as vegetarian. Initially, this was done by simply asking them, “Are you a vegetarian or vegan?” If they answered in the affirmative, they were asked to identify which of the following foods they eat: *Eggs, Milk, Fish or seafood, Chicken or poultry, Meat/red meat occasionally,* or *None of the above*. If they selected *None of the above*, they were asked if they identify as vegan. Participants were asked how long they have been following this way of eating, to which they could select options ranging from *less than a month* to *my whole life.* Adaptive questioning is used in this section so that, for example, if a participant answered “no” to “Are you a vegetarian?” they then moved on to the next question and did not see the questions pertaining to identifying as vegan or how long they have been following that eating pattern. The attitudes and motivations section includes 4 previously validated questionnaires [18-21] (Table 1) that, combined, consist of a total of 81 questions. In this section, questions that ask about similar concepts have been randomly distributed within each of the 4 questionnaires. Responses will be scored according to the published instructions [18-21]. The Dietary Habits section consists of 73 questions from the Dietary Habits Questionnaire that was used in the New Zealand Adult Nutrition Survey 2008/2009, which includes questions about weight loss intentions [22]. Each participant completed the questions from all sections in the same order, and an answer was required for each question. There was no timeframe limitation on completion. Participants were able to go back to previously answered questions and change their answers but there was no review step, and once the questionnaire was completed, they could not access it again. However, participants could leave the questionnaire at any point without completing it. A code was provided to participants so they could log back in and complete the questionnaire later, and reminders were sent to encourage them to do this. Incomplete questionnaires will be included in analysis on a case-by-case basis, depending on the outcome of interest.

**Table 1:** Summary of outcome measures to be collected in the SuNDiAL Project.

Outcome				Assessment Method
**Online Questionnaire**				
	Demographics and health status		Self-report	
	Vegetarian/vegan status		Self-report	
	Dietary Habits		Dietary Habits Questionnaire	
	Attitudes and motivations towards food choice		Rationalizing meat consumption: The 4Ns Questionnaire [19]	
			The Food Choice Questionnaire [21] Ethical Food Choice Motives [18]	
			Dietarian Identity Questionnaire [20]	
**School visit**				
	Estimated usual dietary intake		Two 24-hour recalls, with adjustment of usual intake using MSM^a^	
	Height		Stadiometer	
	Weight		Body weight scales	
	Ulna Length		Steel measuring tape	
**Blood sample**				
	Hemoglobin		Cyanide-free photometry	
	Plasma ferritin		Immunoassay	
	Soluble transferrin receptor		Immunoassay	
	C-reactive protein		Immunoassay	
	Alpha-glycoprotein		Immunoassay	
	Zinc		ICP-MS^b^	
	Selenium		ICP-MS	
	Vitamin B12		Electrochemiluminescence immunoassay	
	Folate		Microbiological Assay	
**Urine Sample**				
	Iodine		ICP-MS	
**Accelerometry**				
	Average daily 24 h Activity		ActiGraph GT3x+, and accompanying wear time and sleep diary.	
		Average daily sleep		
		Average daily sedentary time		
		Average daily light intensity activity		
		Average daily moderate to vigorous intensity physical activity		

^a^MSM: Multiple source method

^b^ICP-MS: Inductively couple plasma mass spectrometry

#### Usual Dietary Intake

Dietary intake was assessed using two 24-hour diet recalls. The first recall was completed face-to-face by a Master of Dietetics student during the in-school data collection visit. The recall was performed using a multiple-pass technique. In the first pass, a “quick list” of all foods and beverages consumed during the previous day (midnight to midnight) is obtained. In the second pass, a detailed description is added to each food and beverage, including cooking methods, recipe information (where appropriate), and brand and product information. In the third pass, the amounts of each food and beverage consumed are obtained. Participants were asked to estimate the amount consumed for each food and beverage using standard household measures (cups, tablespoons, etc), food photographs, shape dimensions, food portion assessment aids (dried beans), and information from packaging. Finally, the full food list was reviewed and any additions or changes were recorded. Upon completion of the recall, participants were asked if salt was added to any of the food consumed, and if so, whether it was iodized. A second recall was completed over video call on a nonconsecutive day, with preference given, where possible, to performing the second recall on a weekend day. All 24-hour diet recalls were entered into FoodWorks dietary analysis software (version 9, Xyris Software) using the New Zealand Food Composition Database, FOODfiles (2016; The New Zealand Institute for Plant and Food Research Limited and the Ministry of Health) and nutrient data for commonly consumed recipes collated in the 2008/09 New Zealand Adult Nutrition Survey [22]. Dietary intake estimated for each nutrient of interest will be adjusted to represent usual intakes based on the estimated within-person variance of vegetarians and nonvegetarians using the Multiple Source Method [23]. Individual daily intakes from supplements will then be calculated and added to the usual intakes. The median (IQR) (for data that are not normally distributed) or mean (SD) (for data that are normally distributed) of daily intakes of energy and key macro- and micro-nutrients, and the main food sources of these nutrients will then be calculated. Molar ratios of phytate:zinc will be calculated to provide estimates of absorbable zinc. The estimated average requirement (EAR) cut-point method will be applied to the usual intake distribution to assess the prevalence of inadequate intakes with the exception of iron, for which the full probability approach will be used because of the skewed iron requirements as a result of menstruation in this population. [24].

#### Anthropometric Assessments

Body weight was measured to the nearest 0.1 kg using calibrated body weight scales. Standing height was measured to the nearest 0.1 cm using a calibrated stadiometer and standardized protocols. Both these measurements were taken with participants wearing light clothing and no footwear. Ulna length was measured on the nondominant arm between the point of the elbow and the midpoint of the prominent bone of the wrist, using a nonexpandable steel measuring tape, with the arm positioned across the torso with the hand resting on the front of the opposite shoulder. Wrist watches and jewelry were removed for this measurement. All anthropometric measurements were performed in duplicate, with a third measurement performed if the difference between the initial two measurements was ≥0.5 units, and the mean of the two closest measurements used as the “true” value. BMI was calculated by weight (in kg) divided by height (in m) squared. BMI z-scores for age and sex will be calculated using the World Health Organization child growth standards [25].

#### Biochemical Assessment

Participants were able to opt-out of providing the blood and urine samples while still participating in the other components. A nonfasting venous blood sample was collected by a trained phlebotomist, using trace element free equipment. A spot urine sample was also collected. Time of collection and time of the last meal were recorded, and all blood and urine samples were transferred in a cooler to an accredited testing laboratory where hemoglobin and vitamin B12 concentrations were analyzed within 8 hours of collection. The remaining blood sample was centrifuged and the serum aliquoted and frozen at –80°C. Frozen serum and urine samples were transferred on ice to the Department of Human Nutrition at the University of Otago where they are stored for later analysis (outlined in Table 1).

#### Twenty-four–hour Activity

Average daily 24-hour activity (sleep, sedentary time, light activity, moderate-to-vigorous physical activity) was measured via a triaxial GT3x+ accelerometer (ActiGraph) among those who consented to accelerometry. Participants were asked to wear the accelerometer continuously for 7 days (except for water-based activities or during full contact sports) on an elasticated belt around their waist, so that the accelerometer was situated over their right hip. The raw accelerometer data were collected at 30 Hz. A daily wear time diary was used to record bedtime, sleep, and wake times and any times when the device was removed. If the device was removed for the purpose of engaging in water-based physical activity or full contact sports, then participants were asked to record the duration and intensity of this activity. Customized Stata (Release 16; StataCorp) code will be used for both accelerometer and log data, to differentiate nonwear and wear time. Time spent asleep will be identified using the Sadeh algorithm [26], and time spent in sedentary behavior and in light intensity and moderate-to-vigorous intensity physical activity will be identified using Freedson cut points [27].

### Statistical Analysis

Statistical analyses will be carried out using Stata (StataCorp). School clusters will be accounted for in all analyses using appropriate methodology (for example, with a sandwich estimator or as a random effect). Estimates of prevalence and means will be reported with 95% confidence intervals. A binary variable for vegetarianism will be created, and comparisons between vegetarians and nonvegetarians will be carried out using regression models: linear regression for continuous outcomes and logistic regression for binary outcomes.

## Results

Recruitment and data collection were conducted and completed with 290 participants in 2019. Data are currently being cleaned and analyzed, with publication of the main results anticipated at the end of 2020.

## Discussion

Anecdotal reports suggest that the popularity of plant-based and vegetarian eating patterns may be rising in parallel with growing concerns about environmental sustainability. This pattern of eating is associated with some positive health outcomes [2,3,9]. Nonetheless, without careful planning, a vegetarian diet can increase the risk of inadequate intakes of bioavailable iron, zinc, calcium, and B vitamins [9,28]. The pubertal growth spurt combined with sexual maturation [12] increases the requirements for these nutrients, and therefore, we propose that female adolescents adopting a vegetarian diet may be at particular risk of nutrient inadequacy and deficiency.

The SuNDiAL project will provide a well-timed investigation into the dietary intakes, micronutrient status, physical activity, motivations, and beliefs of New Zealand adolescent girls. This project will also assess whether the current vegetarian diet consumed by adolescent girls in New Zealand offers substantial benefits or risks over a nonvegetarian eating pattern. The collection of biochemical data, dietary intakes, and 24-hour activity patterns will provide additional important details on benefits associated with vegetarianism, and the identification of individuals “at risk.” The results of this study will support the development of targeted interventions and recommendations aimed at enhancing the health, growth, and development of adolescent girls.

## Abbreviations

REDCap: Research electronic data capture
